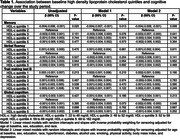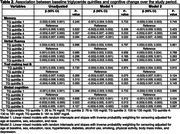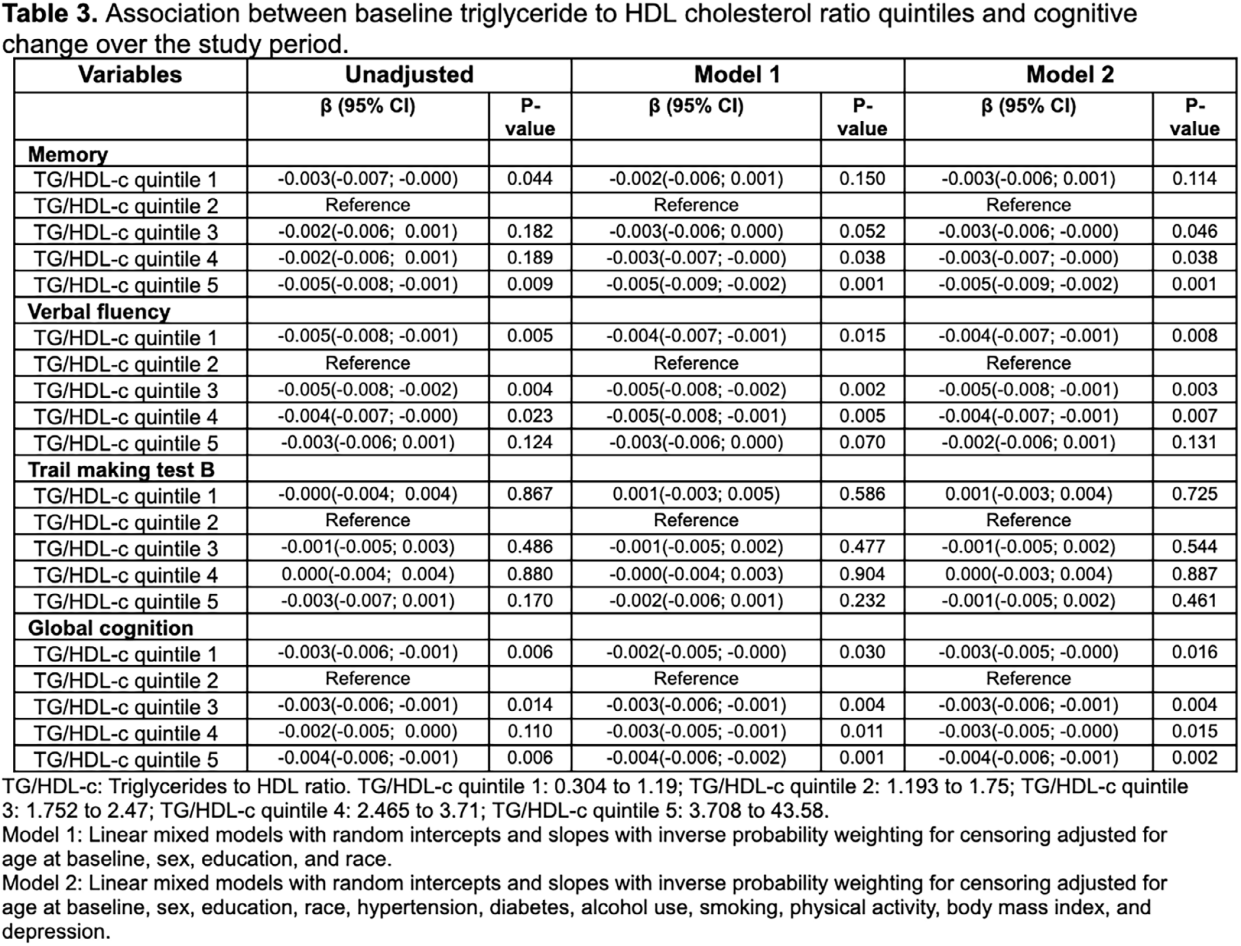# Association of cholesterol, triglycerides, and triglyceride to HDL cholesterol ratio with cognitive decline

**DOI:** 10.1002/alz.092299

**Published:** 2025-01-09

**Authors:** Naomi Vidal‐Ferreira, Marcio S Bittencourt, Isabela M Bensenor, Paulo A Lotufo, Claudia Kimie Suemoto

**Affiliations:** ^1^ University of Sao Paulo Medical School, Sao Paulo Brazil; ^2^ Center for Clinical and Epidemiological Research, Hospital Universitario, University of Sao Paulo, Sao Paulo, Brazil, Sao Paulo Brazil; ^3^ University of São Paulo Medical School, SÃO PAULO Brazil; ^4^ Center for Clinical and Epidemiological Research, Hospital Universitário, University of Sao Paulo, São Paulo, SP, Brazil, Sao Paulo Brazil; ^5^ Division of Geriatrics, Department of Internal Medicine, University of Sao Paulo Medical School, São Paulo, São Paulo Brazil; ^6^ Biobank for Aging Studies of the University of São Paulo, São Paulo Brazil

## Abstract

**Background:**

The association of serum cholesterol with cognitive performance is controversial. Besides, little is known about the association of triglycerides to HDL cholesterol ratio (TG/HDL‐c) with cognitive performance. We aimed to verify the association of baseline cholesterol, triglyceride, and TG/HDL‐c levels with cognitive decline in the Brazilian Longitudinal Study of Adult Health (ELSA‐Brasil) during eight years of follow‐up.

**Method:**

We collected baseline fasting total cholesterol (TC), LDL‐cholesterol (LDL‐c), HDL‐cholesterol (HDL‐c), triglycerides (TG), and computed non‐HDL (TC minus HDL‐c) and TG/HDL‐c (triglycerides divided by HDL‐c). Cognition was assessed in three waves four years apart using the CERAD word list, semantic and phonemic verbal fluency, Trail Making Test B (TMT‐B), and a global composite score. We used inverse probability weighting to account for attrition bias and linear mixed models to investigate the association between lipids and cognition.

**Result:**

In 12,803 participants at baseline, mean age was 51.4±8.87, 55% were women, and 43% were black. After a median follow‐up of 8.2 (7.9‐8.5) years, compared to the second quintile, lower (first quintile) and higher HDL‐c levels were associated with faster memory decline, while higher HDL‐c levels were related to faster global cognitive decline (Table 1). Compared to the second quintile, higher levels of TG were associated with faster memory and global cognitive decline (Table 2). Compared to the second quintile, higher levels of TG/HDL‐c were associated with faster memory decline, while lower (first quintile) and higher levels of TG/HDL‐c were related to faster verbal fluency and global cognitive decline (Table 3). TC, LDL‐c, and non‐HDL were not associated with cognitive decline.

**Conclusion:**

After eight years of follow‐up, HDL‐c, triglycerides, and TG/HDL‐c levels were associated with global and domain‐specific cognitive decline.